# Drugs, clocks and exercise in ageing: hype and hope, fact and fiction

**DOI:** 10.1113/JP282887

**Published:** 2022-10-01

**Authors:** Regula Furrer, Christoph Handschin

**Affiliations:** Biozentrum, University of Basel, Basel, Switzerland

**Keywords:** ageing, exercise, healthspan, lifespan, longevity, rapamycin, reprogramming, rejuvenation, senolytics

## Abstract

Ageing is a biological process that is linked to a functional decline, ultimately resulting in death. Large interindividual differences exist in terms of life- and healthspan, representing life expectancy and the number of years spent in the absence of major diseases, respectively. The genetic and molecular mechanisms that are involved in the regulation of the ageing process, and those that render age the main risk factor for many diseases are still poorly understood.

Nevertheless, a growing number of compounds have been put forward to affect this process. However, for scientists and laypeople alike, it is difficult to separate fact from fiction, and hype from hope. In this review, we discuss the currently pursued pharmacological anti-ageing approaches. These are compared to non-pharmacological interventions, some of which confer powerful effects on health and well-being, in particular an active lifestyle and exercise. Moreover, functional parameters and biological clocks as well as other molecular marks are compared in terms of predictive power of morbidity and mortality. Then, conceptual aspects and roadblocks in the development of anti-ageing drugs are outlined. Finally, an overview on current and future strategies to mitigate age-related pathologies and the extension of life- and healthspan is provided.

## Graphical abstract

**Figure:**
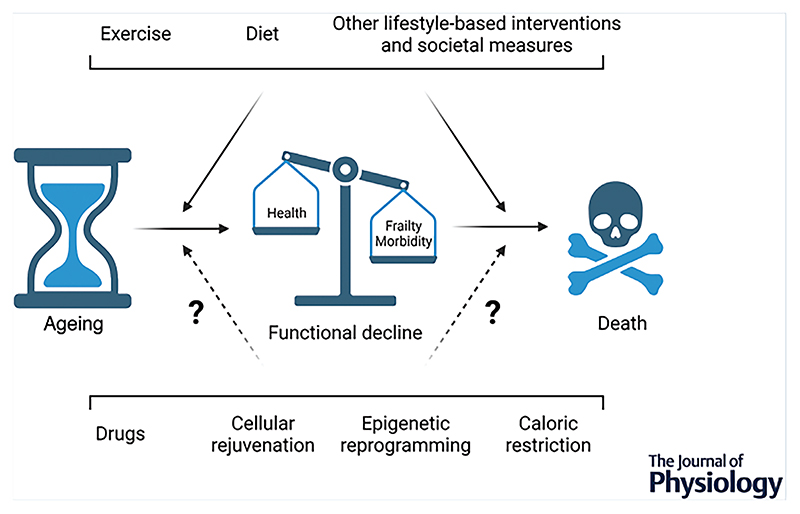
Abstract figure legend

Modulation of the human ageing process. Ageing leads to a functional decline with increased frailty and morbidity, ultimately resulting in death. This process is affected by the genetic set-up, epigenetic pre-disposition and random events, leading to large interindividual differences in regard to health, well-being and life expectancy. Exercise, diet and other lifestyle-based interventions as well as societal measures can help to maximize the chance for healthy ageing. Proposed drugs, cellular, epigenetic and other non-pharmacological approaches such as caloric restriction await validation for safe and efficacious application in human geroprotection.

## Introduction

In all higher organisms, ageing is an inescapable process ultimately resulting in death. In evolutionary terms, removing individuals from the population after the reproductive phase might present an advantage in light of limited food and other resources. Nevertheless, it is unclear whether human ageing represents an active, controlled pathway or is a byproduct of lack of maintenance pressure after reproduction (Kyriazis, 2020). The uncertainty about and the fear of death, coupled witha steady functional deterioration and related health consequences in ageing thus led to endeavours to restore youth or even vanquish mortality throughout human history. Such feats as the search for the Holy Grail, the fountain of youth or the philosopher’s stone are amply documented in myths and fables in almost all human cultures. In modern times, the biology of ageing is the object of intense scientific scrutiny. However, the molecular mechanisms that are involved in ageing remain poorly defined and understood, hinting at a pleiotropic, multifactorial and highly complex sequence of events. Several pathways and processes have been linked to ageing, and proposed as ‘hallmarks’ (Lopez-Otin et al., 2013) or ‘pillars’ (Kennedy et al., 2014), but caveats have been raised and general applicability remains debated (Gems & de Magalhaes, 2021; Keshavarz et al., 2022). Reproducible and robust interventions not only would have the potential to slow down ageing progression, but also reduce the risk for many age-associated chronic diseases (Partridge et al., 2020). A number of these pathologies could be ameliorated by one instead of multiple segregated approaches, thereby overcoming the slow progression in finding directed therapies for some of these, for example neurodegenerative pathologies including dementia, Alzheimer’s or Parkinson’s disease.

### Human and animal models to study ageing

Studies of naturally occurring ‘mutants’, that is centenarians with an extraordinarily long lifespan and, in most cases, an above average number of years in good health (‘healthspan’), are hampered by the small numbers of such individuals (approx. 0.01% of the US population) (Melzer et al., 2020). The identification of more than 50 genetic loci associated with longevity with small effect size underlines the putative multifactorial and polygenic aspects of ageing. Moreover, many of these genetic variants have been linked to the risk for developing a number of chronic diseases, the pre-valence of which increases with age. It thus is unclear whether the genetic polymorphisms that have been described so far directly pertain to the ageing process, or help to stave off age-associated pathologies. Other human studies have been performed in patients suffering from progerias, in which symptoms are observed that resemble an accelerated ageing phenotype (Melzer et al., 2020). Three prototypical forms of progeria, Werner, Hutchinson–Gilford and Cockayne syndrome, are caused by mutations in genes encoding proteins involved in DNA repair, fidelity of DNA replication and/or nuclear architecture (Coppede, 2021). Similarly, mutations in the Werner gene involved in genomic maintenance and DNA repair lead to adult-onset Werner syndrome. However, whether these pathologies represent bona fide accelerated ageing remains debatable, in particular in light of an average lifespan of around 15 years in childhood progeria. Thus, to circumvent the limitations imposed by small populations, diseases with questionable relevance for normal human ageing, and the necessity to study human ageing over years to decades, geroscience to a large extent relies on model organisms, including *Saccharomyces cerevisiae* (yeast), *Drosophila melanogaster* (fruit fly), *Caenorhabditis elegans* (a nematode), rodents and, albeit much rarer, non-human primates.

**Figure 1:**
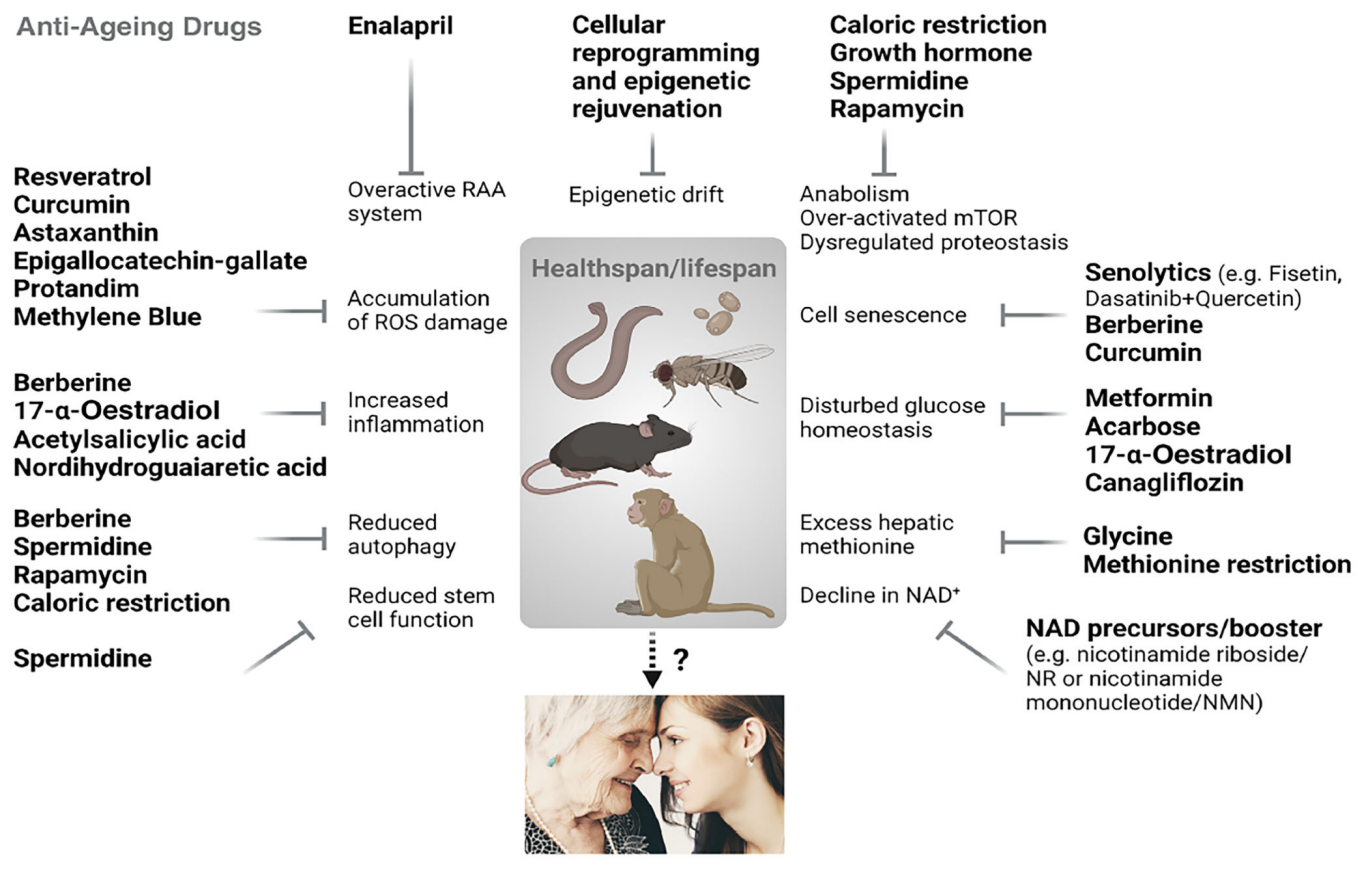
Putative anti-ageing drugs and some of their proposed targets Many natural and synthetic compounds have been proposed to extend lifespan in model organisms, and thus act as potential anti-ageing drugs in humans. Notably, however, most of these compounds failed to do this reproducibly, exhibiting profoundly varying effects in different species, contexts and sexes, and all of them still await human validation in clinical trials. Moreover, for most of these interventions, tolerability, safety and adverse effects are unknown, in particular in long-term administration as would be required for anti-ageing therapy. Finally, it is unclear whether the proposed molecular targets, pathways and mechanisms are indeed dysregulated in human ageing, and if sex-, tissue- and cell type-specific differences exist. Abbreviations: mTOR: mammalian target of rapamycin; RAA, renin–angiotensin–aldosterone; ROS, reactive oxygen species. Image: from Freepik.com (created by Racool_studio). Created with BioRender.com.

Numerous genes, pathways and processes have been identified that affect lifespan of various species. Notably, most monogenic mutations that evoke an extension in lifespan in these model organisms affect proteins involved in anabolic metabolism (e.g. insulin, insulin-like growth factor 1 (IGF-1) and mammalian target of rapamycin (mTOR) signalling) and growth (e.g. pituitary and growth hormone signalling), or reproduction (e.g. related to fertility and fecundity) (Gems & Partridge, 2013). To a large extent, these insights have fuelled a growing pipeline of compounds to potentially affect the ageing process in humans (Fig. 1) (Raffaele & Vinciguerra, 2022). However, since many of these compounds failed to consistently evoke effects in different species, mouse lines, dietary and other contexts, or even both sexes of the same strain, systematic and standardized attempts to study life-extending effects of dietary and pharmacological interventions were initiated by the National Institute on Aging in the Interventions Testing Program (ITP) (Nadon et al., 2017). However, care has to be taken to extrapolate findings from model organisms to humans. First, many of these organisms have unique traits affecting longevity that are absent or at least disputable in humans (e.g. formation of spores in lower organisms, the Dauer stage in nematodes, torpor in mice, or reproduction–lifespan trade-offs in fruit flies). Second, many of the age-expanding mutations discovered in such organisms also confer physiological and functional disadvantages (such as decreased neuromuscular function in the methuselah fly), or fail to be associated with a longevity phenotype in humans (for example in dwarf syndromes with mutations in the same growth-related genes as in mice) (Bartke, 2021). Third, laboratory organisms are studied under tightly controlled genetic and environmental conditions and constraints, in many cases failing to reproduce the complex environment of human daily life. Examples include pathogen-limited or -free rodent breeding and housing stations, in which infections, cancers or other pathological events often result in removal of the respective animals from experimental cohorts, specific *ad libitum* diets, exposure to temperatures below thermoneutrality, or a severely sedentary activity pattern. Fourth, the lifespan of yeast (approx. 14 days, representing chronological and non-dividing, not replicative ageing), *C. elegans* (approx. 20 days), *D. melanogaster* (approx. 2−3 months), *Mus musculus* (approx. 2−3 years), *Rattus norvegicus* (approx. 3−4 years) or *Macaca mulatta* (rhesus monkey, approx. 27−40 years) are far from that achieved in humans (world-wide average life expectancy of approx. 73 years, but over 80 years in a number of countries for both sexes). In fact, based on the number of cortical neurons, and compared to body size, humans might already be long-lived (Herculano-Houzel, 2019; Austad, 2010). Besides the biological considerations of these differences, the much longer lifespan of humans also poses a markedly higher hurdle on tolerability, safety, adverse effects, development of tolerance, evasion or feedback mechanisms of chronic treatments and interventions, which might not be of importance or even observed in short-lived model organisms.

### Pharmacological and non-pharmacological approaches proposed, but not proven to slow down human ageing

Based on these caveats, it is not surprising that many pharmacological and interventional approaches have failed to live up to initial reports and promises (Keshavarz et al., 2022) (Fig. 1). For example, for the polyphenolic compound resveratrol, there is strong evidence that it does not directly bind to the proposed target, sirtuin 1 (SIRT1); many of the effects are only observed in specific mouse strains at very high doses and are dependent on co-administration of a high fat diet; and effects in human clinical trials were small or non-existent (Furrer & Handschin, 2020; Handschin, 2016). Accordingly, clinical development of resveratrol derivatives was stopped 5 years after the acquisition of Sirtris Pharmaceuticals by GlaxoSmithKline in 2008, despite the 720 million US$ price tag. Currently, metformin and rapamycin belong to the most discussed drugs to be repurposed for anti-ageing treatments (Blagosklonny, 2021; Campisi et al., 2019). Clinical trials are planned for these drugs to assess geroprotective and lifespan-enhancing properties. However, in addition to mixed outcomes on mortality in humans or animal models (metformin) (Blagosklonny, 2021; Mohammed et al., 2021), and the immuno-suppressing properties at least at some concentrations (rapamycin), other potential adverse effects have been reported. Importantly, potential beneficial anti-ageing effects would have to outweigh the restrictions imposed on the many benefits of physical activity and other life style-based interventions. Metformin seems to consistently reduce the effects of endurance and resistance training in humans, similar to what has been reported previously for resveratrol (e.g. see Gliemann et al., 2013; Handschin, 2016; Konopka et al., 2019; Kulkarni et al., 2020; Walton et al., 2019). Likewise, rapamycin-induced inhibition of mTOR blunts muscle hypertrophy in different contexts of muscle unloading and reloading in animal models (Morley, 2016). Unfortunately, the metformin and rapamycin studies in model organisms did not include an exercise arm. Such effects thus could not be assessed, and might be of limited relevance in sedentary animals. However, this outcome is of high significance in the human population and is inadequately modelled by caged mice (Booth & Laye, 2009). Other adverse effects of these and other drugs will also have to be identified and evaluated in long-term treatment of humans, as indicated by the potential of mTOR inhibition to affect *β*-amyloid plaques and the development of Alzheimer’s disease (Shi et al., 2022). Although often neglected as an anti-ageing drug, aspirin is another example of a reproducible compound in longevity studies (Campisi et al., 2019), with controversial outcomes on all-cause mortality in healthy elderly (McNeil et al., 2018). Of note, the exact identity, pharmacokinetics, tissue distribution, target mechanism and other crucial pharmacological parameters of many of the proposed anti-ageing compounds are not clear. Similarly, dosing, timing, age of initiation of treatment and other considerations will have to be elucidated (Nelson et al., 2017).

Failures, controversies and question marks are, however, not limited to proposed pharmacological anti-ageing interventions. Caloric restriction robustly extends lifespan in various laboratory models, albeit with decreasing relative payoff when moving from simple to more complex, higher organisms (Dakic et al., 2022; Sohal & Forster, 2014). Notably, in rodents, sex- and strain-specific effects of caloric restriction were reported, which, in some strains, even had a negative effect on lifespan. Thus, the genetic background, time-of-day of feeding, nutrient composition, age at initiation of caloric restriction, extent of caloric restriction and other parameters seem to affect the outcome in rodent, non-human primate and human studies (Campisi et al., 2019; Dakic et al., 2022; Sohal & Forster, 2014). Moreover, questions about adequate controls, and thus the baseline of caloric intake, have been raised. *Ad libitum* feeding might be detrimental to health in control groups of rodents and primates, and the health benefits of caloric restriction in part caused by the amelioration of overeating and obesity-associated pathologies, distinct from any bona fide pro-lifespan effects. In humans, the epidemiological data clearly show an unhealthy baseline of *ad libitum* food intake in many societies, linked to inadequate physical activity. Furthermore, basal, non-exercise activated thermogenic (NEAT) and activity-linked energy expenditure exhibits high interindividual differences, and for any caloric restriction intervention, baseline values and the targeted restriction would have to be strictly personalized. Finally, implementation might be hampered by psychological factors, and side effects, for example on frailty or cognitive performance, might occur (Hofer et al., 2022).

Cellular reprogramming and epigenetic rejuvenation refer to a collection of approaches attempting to leverage recent insights into tissue differentiation and de-differentiation (de Lima Camillo & Quinlan, 2021). Even though promising results in model organisms have been reported, many limitations and potential adverse effects will have to be overcome before safe application in humans can be envisioned. For example, the choice of reprogramming factors, tissue delivery and control of expression, potential tissue de-differentiation or development of cancers have to be considered, in particular in the time scale of human ageing and treatment (de Lima Camillo & Quinlan, 2021). Moreover, many of the proposed anti-ageing drugs, including metformin, resveratrol and rapamycin, decrease reprogramming efficiency at the concentrations for which life-extending benefits have been reported, described as the age reversal–age extension (Arae) paradox in which one anti-ageing treatment adversely affects another (de Lima Camillo & Quinlan, 2021). As a final example, the usage of senolytics, aiming at the selective destruction of senescent cells, will have to address the biological function of cell senescence in differentiation, regeneration, tissue function maintenance and remodelling, wound healing, prevention of tumourigenesis and other processes (Rhinn et al., 2019; Ring et al., 2022).

### Lifestyle interventions that improve healthspan

**Figure 2:**
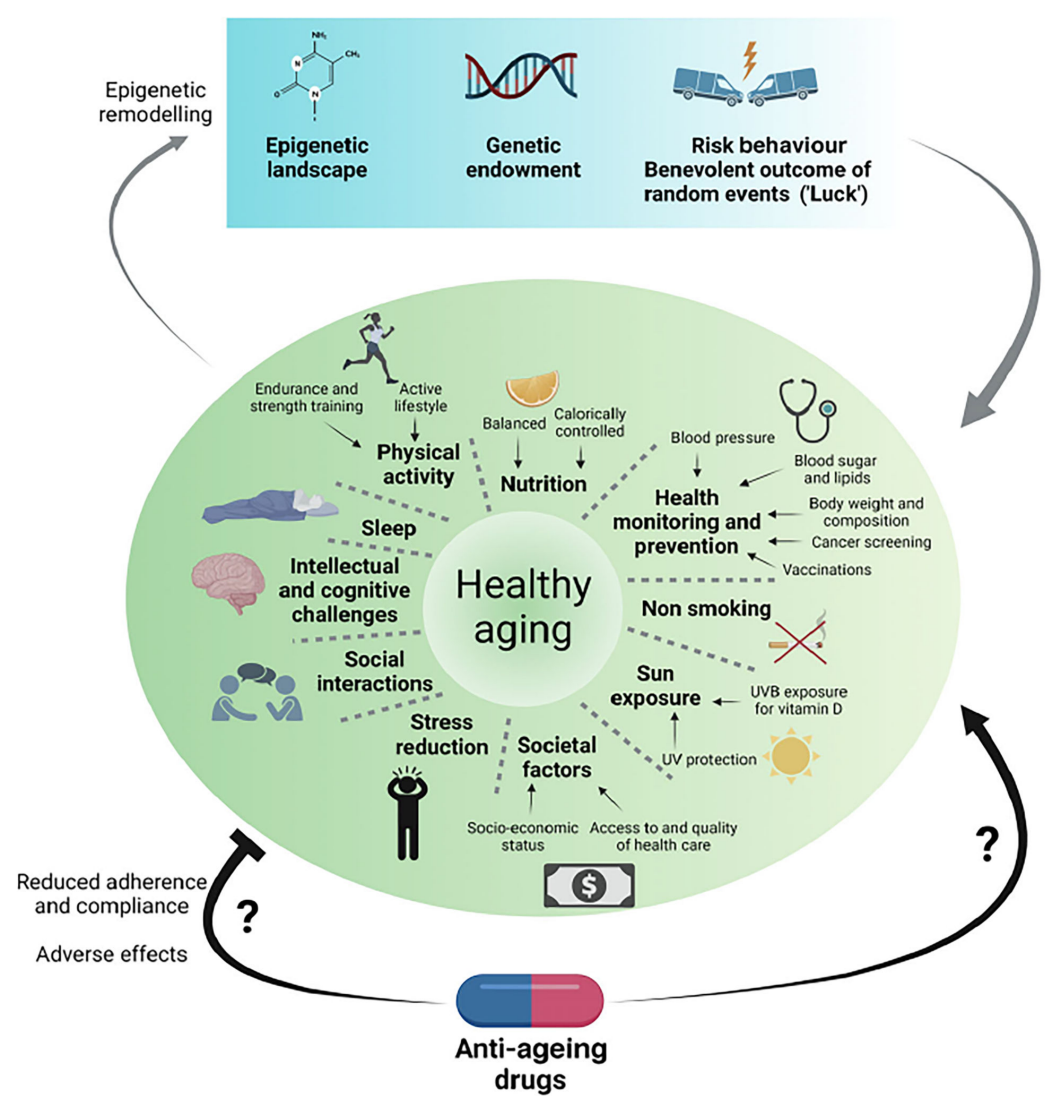
Determinants of healthy ageing Modifiable lifestyle-based interventions have a proven effect on morbidity and mortality in humans. While some of these might affect epigenetic remodelling, the epigenetic drift in ageing, genetic endowment and random events represent age modifiers on which we have minimal or no influence. Novel pharmacological or non-pharmacological interventions could positively affect healthy ageing, but also harbour the potential for adverse effects, and might decrease adherence to and compliance with lifestyle-based approaches. Created with BioRender.com.

In contrast to the yet to be proven potential of these pharmacological and non-pharmacological approaches, exercise provides true, effective robust and reproducible geroprotective effects in model organisms as well as in humans. This is not only achieved by reducing the risk for and incidence of many age-associated chronic diseases, but also by improving quality of life, mean and maximal lifespan (Campisi et al., 2019; Furrer & Handschin, 2020; Garatachea et al., 2015; Goh et al., 2021; Gubert & Hannan, 2021; Handschin, 2016). Importantly, such effects are already reported after moderate activities, and, if performed correctly, training has no adverse effects and outcomes (Booth & Laye, 2009). Physical activity is complemented by other behaviours to affect human health, morbidity and mortality. Collectively, such lifestyle-based interventions currently are the best modalities to promote healthy ageing in humans (Fig. 2), including stress reduction and adequate sleep patterns (Friedman, 2020). Furthermore, a calorically controlled diet balanced in macro- and micronutrients seems to surpass the effects of extreme diet regimens, many of which have been proposed based on experiments in rodents, but with questionable translatability to humans (Lee et al., 2021), for example for low protein-containing diets (Kitada et al., 2019). Then, intellectual and cognitive challenges as well as social interactions help to reduce neuronal declines. Disease prevention, for example in the form of non-smoking and controlled UV exposure, and evidence-based health monitoring and prevention, with proven effectiveness, benefits and minimized risks (Raffle et al., 2019), likewise contribute to health. Societal factors, including access to high quality health care and socio-economic status, are often neglected, albeit highly significant, contributors to life expectancy and health (McMaughan et al., 2020). All of these factors are modifiable by individuals and the society, and have a proven effect on healthy ageing. To a much smaller extent, epigenetic remodelling can be influenced in a targeted manner by exercise or nutrition. Thus, the epigenetic landscape (influenced by hereditability and behaviour), genetic endowment obtained from parents, and the outcome of random events (such as accidents), of which frequency is related to risk behaviour, represent events that affect human lifespan, but on which we have minimal or no influence. The discovery of anti-ageing drugs could have an impact in diverging ways. In the most benevolent manner, such compounds help to promote health up to old age. As outlined above, the use of such drugs might, however, also entail adverse effects, some of which could directly counteract other anti-ageing interventions, for example those elicited by exercise or rejuvenation strategies. Psychologically, the broad availability of anti-ageing drugs could also result in an even further reduction of adherence to and compliance with proven lifestyle-based interventions, now erroneously perceived to be ‘substituted’ by the administration of a pill, with an ultimate net negative outcome on healthy ageing. Overall, the idea of a monotherapy to improve health- and lifespan might still be fiction, and combination polytherapies still years in the making.

### Assessing and monitoring biological age using clocks

**Figure 3:**
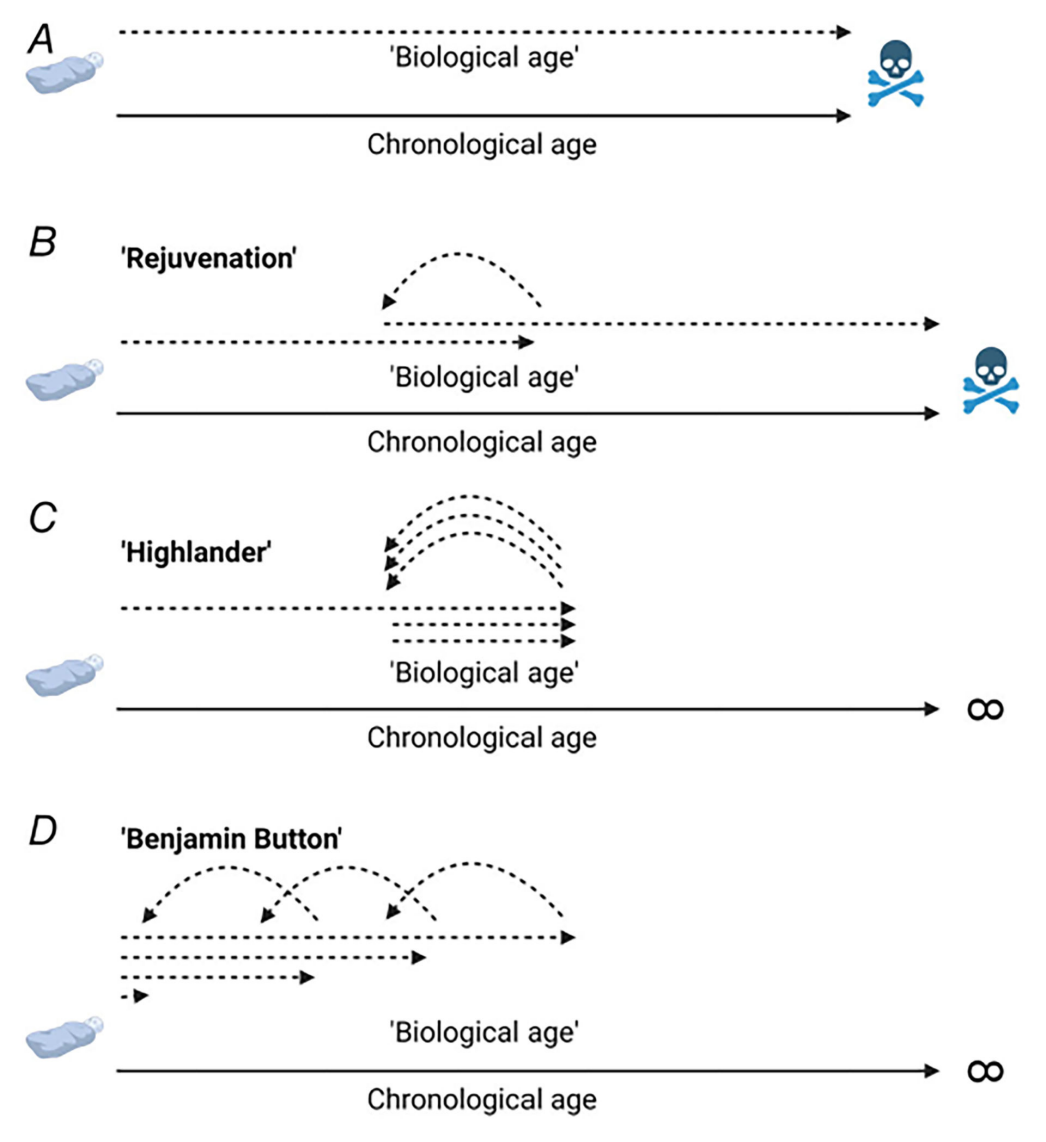
The concept of biological and chronological age *A*, the theoretical concept of a biological age is based on the observation of the vast differences in life-, health- and morbidityspan in individuals. *B*, rejuvenation strategies claim to reduce the biological age, which therefore should result in an increase in chronological age and lifespan, and potentially healthspan. *C*, if rejuvenation strategies indeed would work, and a biological clock accurately represents the relative age of an individual, repeated interventions should result in a ‘Highlander’ effect, effectively pausing chronological ageing. *D*, furthermore, if such rejuvenation interventions were staggered, chronological ageing would be reversed analogous to ‘Benjamin Button’. In both cases, immortality would be achieved. Conceptual and biological arguments for and against such scenarios remain to be formulated and described, respectively. Created with BioRender.com.

The development of potential anti-ageing therapies is hampered by the difficulty for human translation. First, even though hotly debated (Gems, 2015), ageing currently is not classified as a disease by the FDA, EMA and other regulatory agencies, in contrast to many age-associated diseases, for example sarcopenia (ICD-10-CM code M62.84). Therefore, the design and approval of geroprotective clinical trials face high regulatory and ethical hurdles of treating by definition ‘healthy’ individuals, in addition to the logistical and financial problems of following large cohorts over years to decades. Indeed, many of the data on interventions with a proven effect on health- and lifespan, that is exercise, originate from cross-sectional studies, epidemiological assessments, or cohort observations such as meta-analyses of morbidity and mortality of former athletes (Runacres et al., 2021). The necessity for elaborate and costly prospective clinical anti-ageing studies could be mitigated by the use of biomarkers that accurately reflect age and react to treatments in shorter timeframes (Jylhava et al., 2017; Rutledge et al., 2022). In recent years, various molecular biomarkers to benchmark the ‘biological age’, as opposed to the chronological age (Fig. 3A), have been suggested and tested, based on the observation of large interindividual differences in ageing progression, health- and lifespan. Proposed markers include telomere length, transcriptomic profiles, metabolomics age scores, and protein-/glycan-derived age (Jylhava et al., 2017; Rutledge et al., 2022). Most prominently, the changes in the epigenome that occur during development, differentiation and ageing are leveraged to define ‘epigenetic clocks’ (Seale et al., 2022). However, so far, different epigenetic clocks exhibit poor correlation between each other, as well as with functional phenotypes (Fohr et al., 2021; Jylhava et al., 2017). Second, the naked mole rat is an exceptionally long-lived rodent, with very rare occurrences of cancer, cardio-vascular or other age-associated diseases, and thus has been defined as a demographically non-ageing animal, despite clear signs of ‘epigenetic ageing’ (Kerepesi et al., 2022). Furthermore, ageing clocks seem to differ between organs (Nie et al., 2022), in some of which epigenetic clocks provide poor prediction of tissue-specific ageing, for example in skeletal muscle (Sillanpaa et al., 2021). Finally, epigenetic clocks might represent multifactorial composites, in which individual modules are affected differently by perturbations, including epigenetic reprogramming (Levine et al., 2022). Future research will thus not only have to provide further evidence that the molecular basis of different ageing clocks indeed correlates with age, but also that a change in such biomarkers provides meaningful predictive power for phenotypic and functional adaptations. Conceptually, it is finally unclear how the biological compared to a chronological age should be interpreted and applied. Do reported reductions in the biological age, for example after rejuvenation, predict a corresponding increase in chronological age/lifespan (Fig. 3B)? If true, would repetitive rejuvenation eliminate chronological ageing, leading to immortality at a chosen chronological age (as depicted in the ‘Highlander’ movie) (Fig. 3C)? Are there biological constraints to push rejuvenation and hence the biological clock to ever younger ages, leading to a ‘Benjamin Button’-like outcome (Fig. 3D)? Until such questions are resolved on the conceptual and biological levels, reported alterations in a loosely defined biological age, and the differences to chronological age are of limited value.

**Figure 4:**
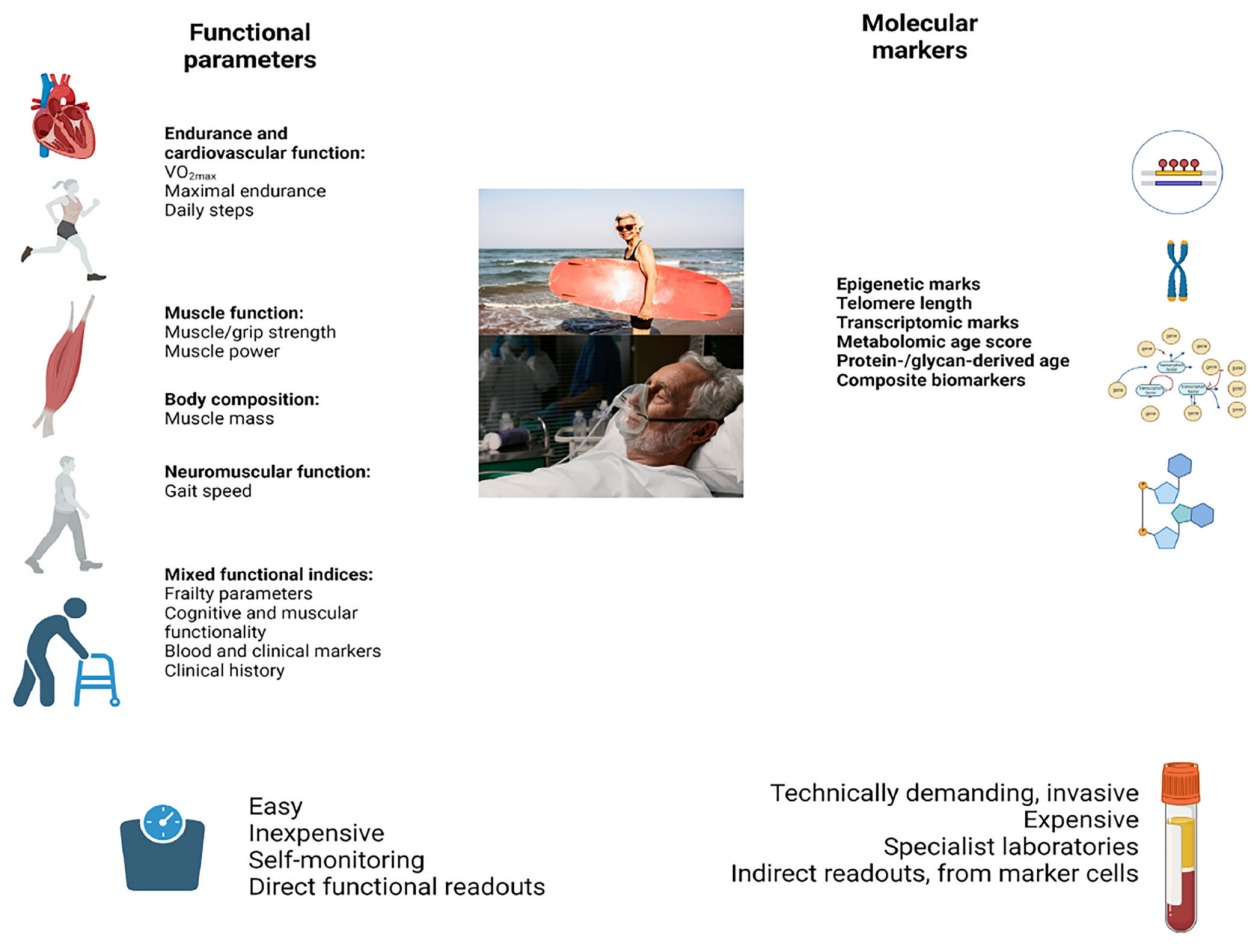
Predictors of ageing, morbidity and mortality Functional parameters, mostly related to neuromuscular capacity, have a high predictive power for human morbidity and mortality. These are easy and inexpensive to measure and implement. Molecular biomarkers are still being refined, and require more invasive collection and specialized analysis. In the future, compound indices that also include diagnostic markers might help to further improve and refine the prediction of healthy ageing. Images: from Freepik.com (created by Rawpixel.com and freepik). Created with BioRender.com.

Intriguingly, ageing biomarkers often prioritize disease risk and health outcomes over bona fide ageing progression, with high potential clinical relevance (Li et al., 2020; Macdonald-Dunlop et al., 2022). In the current state, most molecular clocks are inferior to functional parameters to predict morbidity and mortality in humans (Fig. 4). Endurance and cardiovascular function (for example ${\dot{V}}_{{\text{O}}_{2}\max}$ (Strasser & Burtscher, 2018), maximal endurance (Kodama et al., 2009) or daily steps (Paluch et al., 2022)), skeletal muscle function (for example muscle and grip strength (Sayer & Kirkwood, 2015), muscle power (Losa-Reyna et al., 2022) or muscle mass (Liu et al., 2022)) and neuromuscular function (for example gait speed (White et al., 2013)) all show strong prospective correlation with health. Importantly, these parameters represent at least part of the main decline in functional capacity with age that strongly contributes to loss of independence, hospitalization and admission to nursing homes (Nwagwu et al., 2020). In contrast to the molecular clocks, these biomarkers are inexpensive and easy to determine, provide direct functional readouts, but might lack the mechanistic insights included in the former (Jylhava et al., 2017; Libert et al., 2022; Rutledge et al., 2022; Tzemah-Shahar et al., 2022). Thus, in the future, composite indices that include functional and molecular biomarkers besides diagnostic parameters such as blood pressure, lipids, glucose and others, will improve the determination of health, and the prediction of morbidity and mortality (Libert et al., 2022; Tzemah-Shahar et al., 2022).

## Conclusions and perspectives

**Figure 5:**
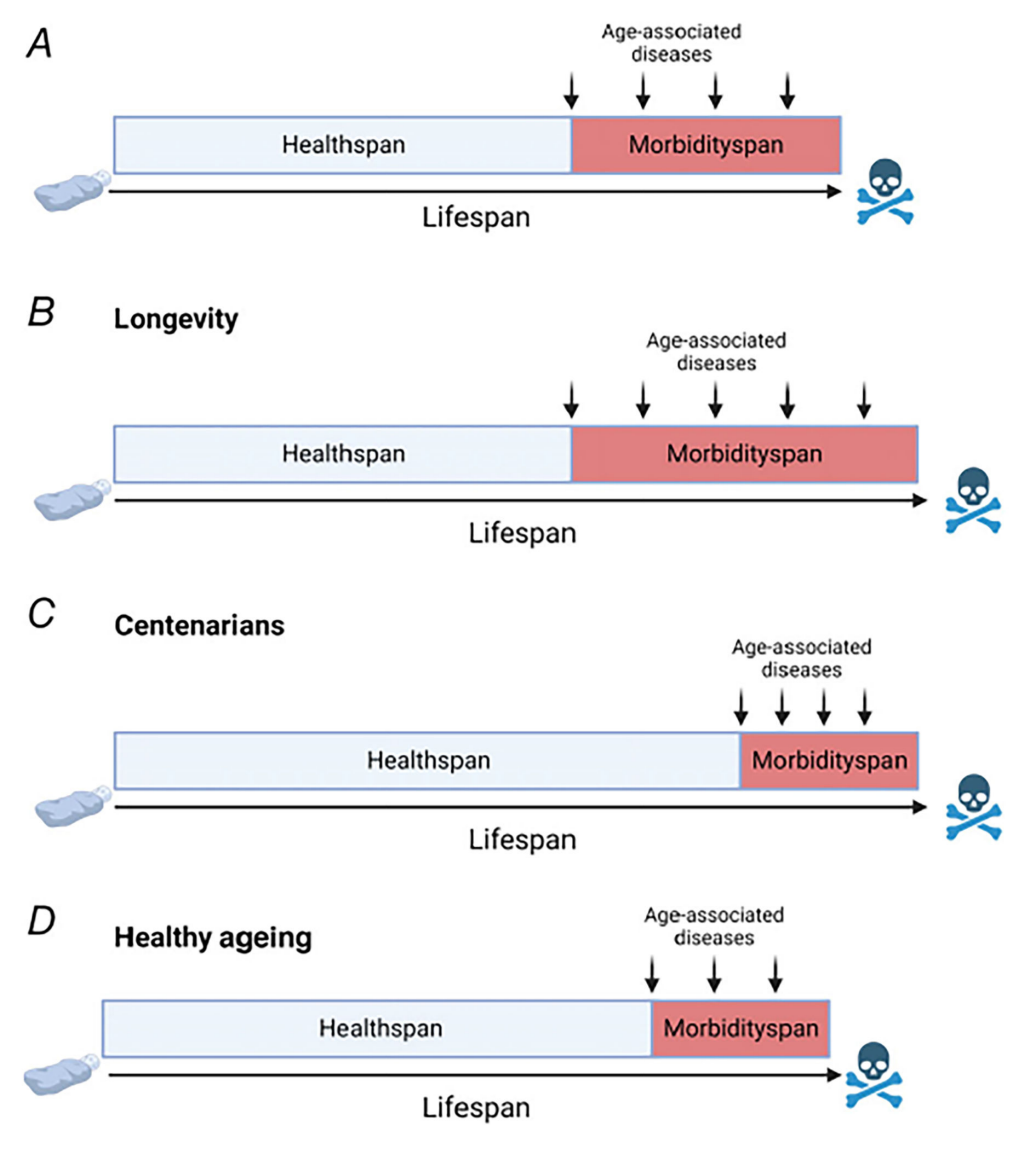
Life-, health- and morbidityspan *A*, lifespan can be divided into healthspan, the number of years spent in good health, and mobidityspan, the number of years in which quality of life is impaired due to age-related functional declines, frailty and the occurrence of an increasing number of age-associated diseases. *B*, anti-ageing strategies aimed at elongation of lifespan are of limited desirability if primarily reached by longer mobidityspans. *C*, centenarians, exceptionally long-lived humans, often have an extended health- and a compressed morbidityspan. *D*, if an upper limit of human lifespan exists, an extension of healthspan, analogous to that in centenarians, should be aimed at to improve quality of life for the individual and relieve health care costs for society. Created with BioRender.com.

Ageing research has undergone a tremendous change in recent decades, and is accelerating at a breathtaking speed due to technological advances, conceptual insights and an increasing availability of financial resources. Nevertheless, due to its multifactorial and complex nature, the mechanistic, cellular, evolutionary and organismal underpinnings of the ageing process are still only rudimentarily understood, in particular in regards to humans. It is not even established whether human lifespan has a limit, or whether future scientific and technological advances will help to break the current ageing records (Colchero et al., 2021; Eisenstein, 2022). Therefore, it is unclear whether the concept of increased lifespan, as reported in model organisms, can be translated to humans, in particular if this would result in an extended morbidity-, but no change in healthspan (Fig. 5A and B). Alternatively, anti-ageing strategies might help to optimize the longevity potential towards a theoretical personal maximum. Optimally, this should be modelled after the long healthspan observed in centenarians (Fig. 5C). However, on the individual as well as the societal level, significant advances would already be achieved if healthspan could be maximized, and morbidityspan minimized without increases in absolute lifespan (Fig. 5D). As long as breakthroughs in novel pharmacological and non-pharmacological approaches are elusive, the current focus should be on proven interventions (Fig. 2). Moreover, these interventions could also help to guide mechanistic studies in geroscience. For example, exercise improves many of the known molecular aspects that have been linked to ageing, including epigenetic marks, mitochondrial function, inflammation, DNA damage or telomere shortening (Garatachea et al., 2015; Goh et al., 2021; Guan & Yan, 2022; Rebelo-Marques et al., 2018; Sellami et al., 2021). Thus, while the influx of funding from individual billionaires facing their own mortality certainly helps to push ageing research, individual and societal measures, for example aimed at promoting physical activity, affordable access to healthy food or balancing socio-economic differences, will have immediate, direct and measurable impacts on our health in old (and young) age.

## Supplementary Material

Additional supporting information can be found online in the Supporting Information section at the end of the HTML view of the article.

Supporting Information
