# Supplementary material for: Drugs, clocks and exercise in ageing: hype and hope, fact and fiction
Source: J Physiol. Author manuscript; Available in PMC 2025 Apr 11. (PMC7617581; doi:10.1113/JP282887)
Supplement: Supporting Information [file EMS204333-supplement-Supporting_Information.pdf]

The Journal of Physiology

Drugs, clocks and exercise in aging: hype and hope, facts and fiction

Regula Furrer and Christoph Handschin
DOI: 10.1113/JP282887

Corresponding author(s): Christoph Handschin (christoph.handschin@unibas.ch)

The following individual(s) involved in review of this submission have agreed to reveal their identity: Robert Vincent Musci (Referee #1); Josiane Broussard (Referee #2)

Review Timeline:

Submission Date:	12-Jul-2022
Editorial Decision:	15-Aug-2022
Revision Received:	15-Aug-2022
Accepted:	25-Aug-2022

Senior Editor: Laura Bennet

Reviewing Editor: Karyn Hamilton

Transaction Report:

(Note: With the exception of the correction of typographical or spelling errors that could be a source of ambiguity, letters and reports are not edited. Depending on transfer agreements, referee reports obtained elsewhere may or may not be included in this compilation. Referee reports are anonymous unless the Referee chooses to sign their reports.)

1st Editorial Decision 15-Aug-2022

Dear Dr Handschin,

Re: JP-SR-2022-282887 "Drugs, clocks and exercise in aging: hype and hope, facts and fiction" by Regula Furrer and Christoph Handschin

Thank you for submitting your invited Review-Symposium to The Journal of Physiology. It has been assessed by a Reviewing Editor and by 2 expert referees and I am pleased to tell you that it is considered to be acceptable for publication following satisfactory revision.

Please advise your co-authors of this decision as soon as possible.

The reports are copied at the end of this email. Please address all of the points and incorporate all requested revisions, or explain in your Response to Referees why a change has not been made.

NEW POLICY: In order to improve the transparency of its peer review process The Journal of Physiology publishes online as supporting information the peer review history of all articles accepted for publication. Readers will have access to decision letters, including all Editors' comments and referee reports, for each version of the manuscript and any author responses to peer review comments. Referees can decide whether or not they wish to be named on the peer review history document.

I hope you will find the comments helpful and have no difficulty in revising your manuscript within 4 weeks.

Your revised manuscript should be submitted online using the links in Author Tasks Link Not Available. This link is to the Corresponding Author's own account, if this will cause any problems when submitting the revised version please contact us.

The image files from the previous version are retained on the system. Please ensure you replace or remove any files that have been revised. Your revised submission should include:

- A Word file of the complete text (including figure legends any Tables);
- An Abstract Figure (with legend in the Article file)
- Each figure as a separate, high quality, file;
- A full Response to Referees;
- A copy of the manuscript with the changes highlighted.
- Author profile. A short biography (no more than 100 words for one author or 150 words in total for two authors) and a portrait photograph of the two leading authors on the paper. These should be uploaded, clearly labelled, with the manuscript submission. Any standard image format for the photograph is acceptable, but the resolution should be at least 300 dpi and preferably more.

You may also upload:

- A 'Cover Art' file for consideration as the Issue's cover image;
- Appropriate Supporting Information (Video, audio or data set https://jp.msubmit.net/cgi-bin/main.plex?form_type=display_requirements#supp).

To create your 'Response to Referees' copy all the reports, including any comments from the Reviewing Editor into a Word, or similar, file and respond to each point in colour or CAPITALS and upload this when you submit your revision.

I look forward to receiving your revised submission.

If you have any queries please reply to this email and staff will be happy to assist.

Yours sincerely,

Professor Laura Bennet
Senior Editor
The Journal of Physiology
https://jp.msubmit.net
http://jp.physoc.org
The Physiological Society
Hodgkin Huxley House
30 Farringdon Lane
London, EC1R 3AW
UK
http://www.physoc.org
http://journals.physoc.org

REQUIRED ITEMS:

-Please upload separate high quality figure files via the submission form.

-Author profile(s) must be uploaded via the submission form. Authors should submit a short biography (no more than 100 words for one author or 150 words in total for two authors) and a portrait photograph of the two leading authors on the paper. These should be uploaded, clearly labelled, with the manuscript submission. Any standard image format for the photograph is acceptable, but the resolution should be at least 300 dpi and preferably more. A group photograph of all authors is also acceptable, providing the biography for the whole group does not exceed 150 words.

EDITOR COMMENTS

Reviewing Editor:

Thank you for submitting this symposium review for consideration by The Journal of Physiology. I've solicited feedback from two expert peer referees. Both referees express enthusiasm for the summary indicating that it is expansive, refreshing, interesting, and timely. Each referee also provides reasonable suggestions for minor revisions to improve clarity and strengthen the arguments put forth in the review. Many of these are editorial in nature. However, some are geared toward improving organization of ideas and supporting arguments with strong citations. I do hope that you will find these careful critiques helpful. Thank you again for submitting this review.

REFEREE COMMENTS

Referee #1:

The authors present an expansive summary of the state of aging research, providing a refreshingly sober perspective of how far the field itself has to go before meaningful lifespan/healthspan extension is achieved through pharmacological intervention.

This reviewer does not have substantial disagreements with the information and arguments presented. However, the reviewer does have a series of comments and suggestions to enhance the quality of the argument presented. A general comment is that the authors do a good job of succinctly describing (in the figure) purported drugs that could improve the healthspan, discusses the benefits of exercise, and introduces the concepts of clocks. They provide a sober perspective in an at times overly optimistic field. However, one suggestion would to explicitly describe interventions/technology that qualify as "fiction" (in their current state) and very briefly highlight that years of work that those areas still require. There are some specific comments below for interventions like CR or fasting and rapamycin. In addition, there are some editorial comments that are meant to only enhance the argument and clarity.

Line 48: The reviewer appreciates the statement that the "molecular mechanisms... remain poorly defined and understood..." and indeed the cited article makes a compelling argument. However, the reviewer would suggest, though, that such a statement might need to be slightly hedged. Arguably, the field has conceptually described physiological declines that occur alongside aging (though these aren't entirely agreed upon). Indeed, the authors present a figure (Fig 1) that attempts to silo the effects of purported/suspected lifespan extending interventions into defined (and perhaps overlapping, but most likely interrelated) processes.

Line 50: From hereon, the paragraph discusses attempts at defining the molecular mechanisms and accurately describes the constraints of studying "mutants" (both long- and short-lived). It may enhance the clarity if the authors were to make this a paragraph on its own.

Line 81: When the authors mention interventions/compounds that "failed to consistently evoke effects" are the authors implying that failure is due to inconsistent treatment regimens? Is that the purpose of contrasting the beginning of that thought with the development of the NIA? What follows after mentioning is another "notion" - that targeting biological aging has the potential to ameliorate a number of chronic diseases as the authors mention. However, in some ways, this seems disorganized. In fact, the reviewer recommends the following sentences be moved to the introduction as it appears to fit

better when discussing the value of geroscience and aging interventions:

"Reproducible and robust interventions not only would have the potential to slow-down aging progression, but also reduce the risk for many age-associated chronic diseases. Thereby, a number of these diseases could be ameliorate by one instead of multiple segregated approaches, and the slow progression in finding directed for some of these... be overcome."

****

Line 102: The reviewer appreciates the four points they raise to caution researchers from extrapolating findings in model organisms to humans. They are well stated. It might be appropriate to change "Finally" to "Fourth" for the sake of consistent listing.

Line 106: The authors bring up a salient point, that humans live much longer than any model organism (thus creating a strong need for model organisms in the first place). Moreover, there is also the argument that humans are already "long-lived" living "4.5 times as expected for their body size."

Austad, S. N. Methusaleh's Zoo: How Nature provides us with Clues for Extending Human Health Span. J Comp Pathol 142, S10-S21 (2010).

This also begs the question of what the value of studying pro-longevity interventions in model organisms that might be "short-lived" (in comparison to their body size-predicted lifespan) for an organism that is already "long-lived" (i.e. humans). In some ways, the value of studying companion animals (particularly dogs) is that many of the limitations of extrapolating findings from model-organisms research (from lines 92 - 104) are circumvented.

Line 120: The reviewer generally agrees that there is good reason to be skeptical that metformin and/or rapamycin will demonstrate positive, geroprotective effects in humans. However, the reviewer does ask for points of clarification:

• Would the authors provide a citation (or citations) demonstrating the mixed outcomes on mortality? Is this a consequence of administering metformin to already unhealthy individuals?

• While rapamycin is immunosuppressive, the reviewer is aware of various manuscripts that suggest that low-doses of rapamycin enhance immune function. (This perhaps raises another difficult question of: even if we identify a drug, we have still have to answer the question: What is the dose? What is the context? What is the frequency?)

Mannick, J. B. et al. mTOR inhibition improves immune function in the elderly. Science Translational Medicine 6, 268ra179-268ra179 (2014).

Line 126: Kulkarni et al discusses the differences in the transcriptomic responses to metformin and resistance exercise training, the Walton et al citation highlights the blunting of functional adaptations.

Walton, R. G. et al. Metformin blunts muscle hypertrophy in response to progressive resistance exercise training in older adults: A randomized, double-blind, placebo-controlled, multicenter trial: The MASTERS trial. Aging Cell 18, (2019).

Line 127: Unfortunately, the reviewer does not have access to the Morley manuscript. Do the authors refer to "different contexts" as the anabolic response to feeding or muscle contractions? Interestingly, the reviewer is unaware of that any studies have researched the effect of chronic administration of rapamycin on muscle protein synthesis or hypertrophy. In aged mice, low dose rapamycin improved grip strength and run time, suggesting a potentially positive (perhaps surprising) benefit of rapamycin on muscle strength/function. While the reviewer also focuses on skeletal muscle, there are documented benefits of rapamycin on physiological function.

Alessandro Bitto... Matt Kaeberlein (2016) Transient rapamycin treatment can increase lifespan and healthspan in middle-aged mice eLife 5:e16351

Lines 127 - 129: the reviewer totally agrees that understanding how these drugs interact with heatlhspan promoting interventions such as exercise is essential and must be considered when prescribing them to the general population. In the current state of the review, the topic of exercise has yet to be addressed and so the point of "blunting" the positive effects of exercise as a negative outcome of metformin and rapamycin isn't immediately apparent. However, given the readership of the Journal of Physiology, this might be an irrelevant comment. Perhaps moving the point in lines 129 - 131 earlier could be beneficial.

Line 129: While the reviewer regularly espouses the multitude of benefits of exercise, the reviewer also acknowledges that a paucity of individuals actually adhere to physical activity guidelines. Thus, aren't humans more likely to resemble sedentary animals?

Line 139: The reviewer believes that discussion of "non-pharmacalogical interventions" could be the start of a new paragraph. The present paragraph is already quite long with many different topics.

Line 142: Many of the issues (strain, sex, species- specific effects, as well as time of day, nutrient composition, age of initiation, dose of treatment) of caloric restriction can also apply to rapamycin and/or metformin. To the strengthen the manuscript, would the authors speak to the present challenges in the aging field to determine the dosing, timing, initiation time of drug, etc?

Line 182: The reviewer does not disagree that the listed interventions (sleep, stress reduction, calorie-controlled diet) promote healthy aging. However, it is necessary to provide citations for these statements.

Line 190: What do the authors mean by "evidence-based health monitoring" and prevention? Again, citations are needed for these statements.

Lines 194 - 198: The reviewer is uncertain as to what the authors mean when discussing epigentic remodeling, its landscape, genetic endowment, and random events.

Line 203: Again, the reviewer agrees with the sentiment (that a pill may lead to poorer adherence to beneficial health behaviors), but do the authors have citation that demonstrates this assumption and/or demonstrates a net negative outcome?

Line 240: At the risk of alienating oneself, the reviewer has not seen the Highlander movie, but trusts that this is an appropriately made analogy.

Line 248: The authors make a strong and relevant point here.

Line 254: Will the authors provide a citation to support this statement?

Referee #2:

This is an interesting and timely paper by Furrer and Handschin on the impact of novel factors that influence aging, specifically, drugs, circadian clocks and exercise. I appreciate the critical view the authors give to this topic which has received much hype. The paper is excellently written and compelling.

Minor suggestions are provided to improve the clarity and message of the manuscript.

1. Some of the longer sentences lose the connection between object and become a bit confusing:

"Such feats are exemplified by the search for the Holy Grail, the fountain of youth or the philosopher's stone, amply documented in myths and fables in almost all human cultures."

"Thereby, a number of these diseases could be ameliorated by one instead of multiple segregated approaches, and the slow progression in finding directed therapies for some of these, e.g. neurodegenerative pathologies including dementia, Alzheimer's or Parkinson's disease, be overcome."

2. Consider moving e.g. statements into parentheticals to assist with clarity:

"First, many of these organisms have unique traits affecting longevity that are absent or at least disputable in humans, e.g. formation of spores in lower organisms, the Dauer stage in nematodes, torpor in mice, or reproduction-life span trade-offs in fruit flies. Second, many of the age-expanding mutations discovered in such organisms also confer physiological and functional disadvantages, e.g. decreased neuromuscular function in the methuselah fly, or fail to be associated with a longevity phenotype in humans, for example in dwarf syndromes with mutations in the same growth-related genes as in mice"

3. In line 182, "Therefore, lifestyle-based interventions currently are the best modalities to promote healthy aging in humans" the authors are referring here only to exercise or PA. Consider moving a strong conclusion like this to later in this section after other lifestyle interventions are described or consider adding "exercise" or "physical activity" to the sentence if that is the meaning.

4. The section "Lifestyle interventions that improve healthspan" is somewhat confusing because it combines multiple interventions but hits on exercise very strongly in the beginning, mentions a couple of other lifestyle interventions, and then touches on anti-aging drugs. Consider either fleshing out this section into the areas described, or focus it more on exercise as it is somewhat disjointed and distracting at the moment.

END OF COMMENTS

Reply to the reviewers' comments

We would like to thank both reviewers for the thorough reading and the constructive comments. We now have implemented these points and are convinced that in the revised form, the manuscript has gained in impact and interest for a general readership. As pointed out in the point-by-point response below, our revisions however were confined by the size and reference limitations imposed by the *Journal of Physiology*.

Additional changes that were made (not requested by the reviewers):

-Figure 1 now also includes a depiction of yeast as model organism

-The legend to Figure 1 now explains the abbreviations RAA and ROS

Point-by-point responses:

Reviewer #1 .... page 2

Reviewer #2 .... page 8

Reviewer #1

General comment: A general comment is that the authors do a good job of succinctly describing (in the figure) purported drugs that could improve the healthspan, discusses the benefits of exercise, and introduces the concepts of clocks. They provide a sober perspective in an at times overly optimistic field. However, one suggestion would to explicitly describe interventions/technology that qualify as "fiction" (in their current state) and very briefly highlight that years of work that those areas still require.

Reply: We completely agree with the reviewer that this aspects should be greatly expanded. However, due to the size and reference limitations of the *Journal of Physiology*, such a comprehensive discussion might fit better to a more comprehensive review. Nevertheless, we now have added an additional sentence in the "Lifestyle interventions that improve healthspan" section to mention that monotherapies to address aging and aging-related diseases most certainly are fiction, at least based on the current research and results.

Line 48: The reviewer appreciates the statement that the "molecular mechanisms... remain poorly defined and understood..." and indeed the cited article makes a compelling argument. However, the reviewer would suggest, though, that such a statement might need to be slightly hedged. Arguably, the field has conceptually described physiological declines that occur alongside aging (though these aren't entirely agreed upon). Indeed, the authors present a figure (Fig 1) that attempts to silo the effects of purported/suspected lifespan extending interventions into defined (and perhaps overlapping, but most likely interrelated) processes.

Reply: We agree that we might have described the mechanisms/hallmarks/pillars too imprecisely. We therefore have now added a short discussion of these (including the corresponding references), and the caveats in the "Introduction" section.

Line 50: From hereon, the paragraph discusses attempts at defining the molecular mechanisms and accurately describes the constraints of studying "mutants" (both long- and short-lived). It may enhance the clarity if the authors were to make this a paragraph on its own.

Reply: We have now separated this section into a new, distinct paragraph as suggested. We have furthermore included the first part of the following section into this new paragraph in order to include both human as well as animal models that are used to study aging.

Line 81: When the authors mention interventions/compounds that "failed to consistently evoke effects" are the authors implying that failure is due to inconsistent treatment regimens? Is that the purpose of contrasting the beginning of that thought with the development of the NIA? What follows after mentioning is another "notion" - that targeting biological aging has the potential to ameliorate a number of chronic diseases as the authors mention. However, in some ways, this seems disorganized. In fact, the reviewer recommends the following sentences be moved to the

introduction as it appears to fit better when discussing the value of geroscience and aging interventions: "Reproducible and robust interventions not only would have the potential to slow-down aging progression, but also reduce the risk for many age-associated chronic diseases. Thereby, a number of these diseases could be ameliorate by one instead of multiple segregated approaches, and the slow progression in finding directed for some of these... be overcome."

Reply: The failure in consistent outcomes for many of the compounds that were tested has various and multifactorial reasons, from differences in treatment regimens, times, doses, strain backgrounds, diets and others. To (hopefully) clarify this without having to expand the section too much, we now have added that the ITP initiative aims at standardizing the studies. Second, the sentences have now been moved to the "Introduction" section as proposed.

Line 102: The reviewer appreciates the four points they raise to caution researchers from extrapolating findings in model organisms to humans. They are well stated. It might be appropriate to change "Finally" to "Fourth" for the sake of consistent listing.

Reply: "Finally" has been changed to "Fourth" as suggested.

Line 106: The authors bring up a salient point, that humans live much longer than any model organism (thus creating a strong need for model organisms in the first place). Moreover, there is also the argument that humans are already "long-lived" living "4.5 times as expected for their body size." Austad, S. N. Methusaleh's Zoo: How Nature provides us with Clues for Extending Human Health Span. J Comp Pathol 142, S10-S21 (2010). This also begs the question of what the value of studying pro-longevity interventions in model organisms that might be "short-lived" (in comparison to their body size-predicted lifespan) for an organism that is already "long-lived" (i.e. humans). In some ways, the value of studying companion animals (particularly dogs) is that many of the limitations of extrapolating findings from model-organisms research (from lines 92 - 104) are circumvented.

Reply: This is an excellent point that we have not considered: we now have added this to the manuscript.

Line 120: The reviewer generally agrees that there is good reason to be skeptical that metformin and/or rapamycin will demonstrate positive, geroprotective effects in humans. However, the reviewer does ask for points of clarification: I.) Would the authors provide a citation (or citations) demonstrating the mixed outcomes on mortality? Is this a consequence of administering metformin to already unhealthy individuals? II.) While rapamycin is immunosuppressive, the reviewer is aware of various manuscripts that suggest that low-doses of rapamycin enhance immune function. (This perhaps raises another difficult question of: even if we identify a drug, we have still have to answer the question: What is the dose? What is the context? What is the frequency?) Mannick, J. B. et al. mTOR inhibition improves immune function in the elderly. Science Translational Medicine 6, 268ra179-268ra179 (2014).

Reply: I.) we have now added a reference (PMID 34421827) that summarizes different trials showing that even though in some contexts, metformin decreased mortality, this was not observed universally. In most studies, metformin has assessed in patients, most commonly type 2 diabetics, in most of which a positive effect on mortality has been demonstrated. However, the results are more mixed for other symptoms, e.g. cardiovascular parameters, in which also neutral effects of metformin have been reported. Since studies in healthy individuals are currently ongoing or planned, we now have also added/summarized the mixed results of animal models to the discussion. II.) As pointed out by the reviewer, immune-enhancing effects of rapamycin have been demonstrated. We therefore have added "at least at some concentrations" to the corresponding sentence. At the moment, it however is not clear (at least to us) how this should be brought about: the immune suppressing effect of rapamycin (and its use as an immunosuppressant in the clinic) rely on mTOR inhibition in immune cells. As circulating cells, these would be exposed to rapamycin even more so than tissue-/organ-cells.

Line 126: Kulkarni et al discusses the differences in the transcriptomic responses to metformin and resistance exercise training, the Walton et al citation highlights the blunting of functional adaptations. Walton, R. G. et al. Metformin blunts muscle hypertrophy in response to progressive resistance exercise training in older adults: A randomized, double-blind, placebo-controlled, multicenter trial: The MASTERS trial. Aging Cell 18, (2019).

Reply: We have not added this reference originally due to the limitations imposed by the journal but agree with the reviewer that it is important and therefore have now added this reference..

Line 127: Unfortunately, the reviewer does not have access to the Morley manuscript. Do the authors refer to "different contexts" as the anabolic response to feeding or muscle contractions? Interestingly, the reviewer is unaware of that any studies have researched the effect of chronic administration of rapamycin on muscle protein synthesis or hypertrophy. In aged mice, low dose rapamycin improved grip strength and run time, suggesting a potentially positive (perhaps surprising) benefit of rapamycin on muscle strength/function. While the reviewer also focuses on skeletal muscle, there are documented benefits of rapamycin on physiological function. Alessandro Bitto... Matt Kaeberlein (2016) Transient rapamycin treatment can increase lifespan and healthspan in middle-aged mice eLife 5:e16351

Reply: We now have clarified that most studies demonstrated an inhibitory effect of rapamycin on muscle unloading and reloading in animal models. As mentioned, no data exist about the effect of rapamycin on muscle hypertrophy and strength in resistance training. We also did not want to discount the numerous beneficial effects of rapamycin in aged animals. However, until tested, it is unclear whether these a.) are also seen in humans and b.) would outweigh the potential adverse effects on muscle mass and strength elicited by resistance training. As described in the manuscript, all rapamycin studies in mice have been performed in sedentary animals. There are compelling arguments that regularly caged mice are extremely sedentary and thus not a good model for baseline activity (e.g. see PMID 19723782). We have now clarified this line of thought in the text.

Lines 127 - 129: the reviewer totally agrees that understanding how these drugs interact with heatlhspan promoting interventions such as exercise is essential and must be considered when prescribing them to the general population. In the current state of the review, the topic of exercise has yet to be addressed and so the point of "blunting" the positive effects of exercise as a negative outcome of metformin and rapamycin isn't immediately apparent. However, given the readership of the Journal of Physiology, this might be an irrelevant comment. Perhaps moving the point in lines 129 - 131 earlier could be beneficial.

Reply: We now have moved this sentence to the beginning of the discussion to improve clarity as suggested.

Line 129: While the reviewer regularly espouses the multitude of benefits of exercise, the reviewer also acknowledges that a paucity of individuals actually adhere to physical activity guidelines. Thus, aren't humans more likely to resemble sedentary animals?

Reply: In many regards, this certainly is true. However, as outlined above (and described in PMID 19723782), the small size of home cages, the easy accessibility of food and water, and other factors seem to reduce in-cage activity of mice to a pathological setting that might not reflect human behavior. Moreover, even though plagued by low compliance and adherence, an active lifestyle should be aspired to (and promoted by society, physicians etc): thus, for the individuals that try to implement that, this will become highly relevant.

Line 139: The reviewer believes that discussion of "non-pharmacalogical interventions" could be the start of a new paragraph. The present paragraph is already quite long with many different topics.

Reply: We now have split up the paragraph as suggested.

Line 142: Many of the issues (strain, sex, species- specific effects, as well as time of day, nutrient composition, age of initiation, dose of treatment) of caloric restriction can also apply to rapamycin and/or metformin. To the strengthen the manuscript, would the authors speak to the present challenges in the aging field to determine the dosing, timing, initiation time of drug, etc?

Reply: We now have added a brief discussion of these parameters to the pharmacological paragraph of this section as suggested.

Line 182: The reviewer does not disagree that the listed interventions (sleep, stress reduction, calorie controlled diet) promote healthy aging. However, it is necessary to provide citations for these statements.

Reply: In a more comprehensive review, we would have cited many more references for these statements, but also other sections, e.g. the numerous studies demonstrating the impact of exercise, cardiovascular fitness, muscle strength etc. on mortality. Due to the limitations imposed by the journal, we unfortunately had to restrict ourselves to some representative references. We how have added an additional reference in this section (PMID 33010900) that summarizes the impact of many of the discussed lifestyle factors on healthy aging.

Line 190: What do the authors mean by "evidence-based health monitoring" and prevention? Again, citations are needed for these statements.

Reply: With this statement, we wanted to point towards heath monitoring/screening with a clear benefit, minimized risk, and high effectiveness. We now have cited the book "Screening: Evidence and Practice" as reference to clarify this point.

Lines 194 - 198: The reviewer is uncertain as to what the authors mean when discussing epigenetic remodeling, its landscape, genetic endowment, and random events.

Reply: We would like to have expanded this section, but had to limit the length due to the journal guidelines. We now have tried to clarify that we mean the epigenetic marks inherited from the parents and affected by behavior, the genes inherited from the parents, and random events such as accidents, all of which affect how (and whether) we age.

Line 203: Again, the reviewer agrees with the sentiment (that a pill may lead to poorer adherence to beneficial health behaviors), but do the authors have citation that demonstrates this assumption and/or demonstrates a net negative outcome?

Reply: This is mainly an assumption, however based on other contexts, e.g. so-called "exercise mimetics" (see PMID 19723782), and at least anecdotal evidence in type 2 diabetes and other diseases in which the availability of a drug reduces the perceived necessity for the patient in engaging in lifestyle-based interventions. We have modified the sentence to make this more clear.

Line 240: At the risk of alienating oneself, the reviewer has not seen the Highlander movie, but trusts that this is an appropriately made analogy.

Reply: Even though the movie is past its prime (from the year 1986), it is still high on our recommended list, not least due to its extraordinary soundtrack by Queen. In Highlander, the “immortals” will remain at a certain age forever, at least until killed by another immortal (=> “there can only be one”). We thought that this might provide the correct analogy to the particular model in which repeated rejuvenation will keep an individual at a certain biological age.

Line 248: The authors make a strong and relevant point here.

Reply: We now have added a reference to further highlight this point.

Line 254: Will the authors provide a citation to support this statement?

Reply: We have now added references to support this statement as suggested.

Reviewer #2

1. Some of the longer sentences lose the connection between object and become a bit confusing: "Such feats are exemplified by the search for the Holy Grail, the fountain of youth or the philosopher's stone, amply documented in myths and fables in almost all human cultures." "Thereby, a number of these diseases could be ameliorated by one instead of multiple segregated approaches, and the slow progression in finding directed therapies for some of these, e.g. neurodegenerative pathologies including dementia, Alzheimer's or Parkinson's disease, be overcome."

Reply: We have now amended these sentences as proposed and hope to provide more clarity.

2. Consider moving e.g. statements into parentheticals to assist with clarity: "First, many of these organisms have unique traits affecting longevity that are absent or at least disputable in humans, e.g. formation of spores in lower organisms, the Dauer stage in nematodes, torpor in mice, or reproduction-life span trade-offs in fruit flies. Second, many of the age-expanding mutations discovered in such organisms also confer physiological and functional disadvantages, e.g. decreased neuromuscular function in the methuselah fly, or fail to be associated with a longevity phenotype in humans, for example in dwarf syndromes with mutations in the same growth-related genes as in mice"

Reply: We now have introduced parentheses to improve clarity as suggested.

3. In line 182, "Therefore, lifestyle-based interventions currently are the best modalities to promote healthy aging in humans" the authors are referring here only to exercise or PA. Consider moving a strong conclusion like this to later in this section after other lifestyle interventions are described or consider adding "exercise" or "physical activity" to the sentence if that is the meaning.

Reply: We now have changed the sentence to clarify that we primarily refer to exercise, but will expand lifestyle-based interventions beyond physical activity in the next few sentences.

4. The section "Lifestyle interventions that improve healthspan" is somewhat confusing because it combines multiple interventions but hits on exercise very strongly in the beginning, mentions a couple of other lifestyle interventions, and then touches on anti-aging drugs. Consider either fleshing out this section into the areas described, or focus it more on exercise as it is somewhat disjointed and distracting at the moment.

Reply: In a more comprehensive setting, we would have liked to expand on all of these factors. However, the size and reference limitations imposed by the journal preclude a lengthy discussion.

We now have changed the sentences to make clear that while ample evidence for a role of exercise in healthy aging exist, other behaviors and interventions also contribute, as we tried to summarize in Figure 2. We hope that thereby, more coherence is provided, even though we were not able to flesh out the individual points.

Dear Professor Handschin,

Re: JP-SR-2022-282887R1 "Drugs, clocks and exercise in aging: hype and hope, facts and fiction" by Regula Furrer Christoph Handschin

I am pleased to tell you that your Symposium Review article has been accepted for publication in The Journal of Physiology, subject to any modifications to the text that may be required by the Journal Office to conform to House rules.

NEW POLICY: In order to improve the transparency of its peer review process The Journal of Physiology publishes online as supporting information the peer review history of all articles accepted for publication. Readers will have access to decision letters, including all Editors' comments and referee reports, for each version of the manuscript and any author responses to peer review comments. Referees can decide whether or not they wish to be named on the peer review history document.

The last Word version of the paper submitted will be used by the Production Editors to prepare your proof. When this is ready you will receive an email containing a link to Wiley's Online Proofing System. The proof should be checked and corrected as quickly as possible.

All queries at proof stage should be sent to tjp@wiley.com

The accepted version of the manuscript is the version that will be published online until the copy edited and typeset version is available. Authors should note that it is too late at this point to offer corrections prior to proofing. Major corrections at proof stage, such as changes to figures, will be referred to the Reviewing Editor for approval before they can be incorporated. Only minor changes, such as to style and consistency, should be made a proof stage. Changes that need to be made after proof stage will usually require a formal correction notice.

Are you on Twitter? Once your paper is online, why not share your achievement with your followers. Please tag The Journal (@jphysiol) in any tweets and we will share your accepted paper with our 22,000+ followers!

Yours sincerely,

Professor Laura Bennet
Senior Editor
The Journal of Physiology
https://jp.msubmit.net
http://jp.physoc.org
The Physiological Society
Hodgkin Huxley House
30 Farringdon Lane
London, EC1R 3AW
UK
http://www.physoc.org
http://journals.physoc.org

Comments:

Reviewing Editor:

Thank you so much for your close attention to the previous reviewer critiques. As you will see, both are fully satisfied with the revisions you've made.

Senior Editor:

Thank you for the diligent changes made. This is paper provides great insights into the aging field.

REFEREE COMMENTS:

Referee #1:

The authors adequately addressed all of the previous comments. There are no further comments.

The reviewer appreciates the authors' diligence in the revision, as well as for the movie recommendation.

Referee #2:

The authors have been very responsive to reviews and have increased the clarity of the manuscript as requested.

* IMPORTANT NOTICE ABOUT OPEN ACCESS *

To assist authors whose funding agencies mandate public access to published research findings sooner than 12 months after publication The Journal of Physiology allows authors to pay an open access (OA) fee to have their papers made freely available immediately on publication.

You will receive an email from Wiley with details on how to register or log-in to Wiley Authors Services where you will be able to place an OnlineOpen order.

You can check if you funder or institution has a Wiley Open Access Account here https://authorservices.wiley.com/author-resources/Journal-Authors/licensing-and-open-access/open-access/author-compliance-tool.html

Your article will be made Open Access upon publication, or as soon as payment is received.

If you wish to put your paper on an OA website such as PMC or UKPMC or your institutional repository within 12 months of publication you must pay the open access fee, which covers the cost of publication.

OnlineOpen articles are deposited in PubMed Central (PMC) and PMC mirror sites. Authors of OnlineOpen articles are permitted to post the final, published PDF of their article on a website, institutional repository, or other free public server, immediately on publication.

Note to NIH-funded authors: The Journal of Physiology is published on PMC 12 months after publication, NIH-funded authors DO NOT NEED to pay to publish and DO NOT NEED to post their accepted papers on PMC.

****

1st Confidential Review **15-Aug-2022**
